# Virginity Testing Beyond a Medical Examination

**DOI:** 10.5539/gjhs.v8n7p152

**Published:** 2015-11-18

**Authors:** Mehri Robatjazi, Masoumeh Simbar, Fatemeh Nahidi, Jaber Gharehdaghi, Mohammadali Emamhadi, Abou-Ali Vedadhir, Hamid Alavimajd

**Affiliations:** 1 Departments of Midwifery & Reproductive Health, Shahid Beheshti University of Medical Sciences, Tehran, Iran; 2 Department of Midwifery & Reproductive Health, School of Nursing and Midwifery, Shahid Beheshti University of Medical Sciences, Tehran, Iran; 3 Legal Medicine Research Center, Legal Medicine Organizations, Tehran, Iran; 4 Department of Forensic Medicine, Shahid Beheshti University of Medical Science, Tehran, Iran; 5 Departments of Anthropology, Faculty of Social Sciences, University of Tehran, Tehran, Iran; 6 Department of Biostatistics, Shahid Beheshti University of Medical Sciences, Tehran, Iran

**Keywords:** virginity testing, examiner, perception, experience, interpretive phenomenology analysis

## Abstract

Apart from religious values, virginity is important in different communities because of its prominent role in reducing sexually transmitted diseases and teen pregnancies. Even though virginity testing has been proclaimed an example of violence against women by the World Health Organization, it is still conducted in many countries, including Iran. 16 in-depth, semi-structured interviews were conducted with participants aged 32 to 60 years to elucidate the perceptions and experiences of Iranian examiners of virginity testing.

The perception and experience of examiners were reflected in five main themes. The result of this study indicated that virginity testing is more than a medical examination, considering the cultural factors involved and its overt and covert consequences. In Iran, testing is performed for both formal and informal reasons, and examiners view such testing with ambiguity about the accuracy and certainty of the diagnosis and uncertainty about ethics and reproductive rights. Examiners are affected by the overt and covert consequences of virginity testing, beliefs and cultural values underlying virginity testing, and informal and formal reasons for virginity testing.

## 1. Introduction

Virginity, meaning no sex before marriage for both sexes, is of significant importance in Christianity, Judaism, and Islam (Amy, 2008). Moreover, because of its role in preventing sexually transmitted diseases (STDs) and unplanned pregnancies, the United States has planned virginity pledge programs at an annual cost of $200 million to encourage boys and girls to preserve their virginity with sexual abstinence until marriage (Antoinette, Landor, & Gordon, 2013).

Virginity testing is also very important in assessing sexual assault (Tofighi Zavareh, Mosavipour, & Nematolahi, 2002). In recent decades, colposcopy (Hobbs & Wynne, 1996) and telemedicine applications have been used to increase the accuracy and reliability of virginity testing, especially in child victims of rape (MacLeod et al., 2009). Tofighi-Zavareh and Hejazi also emphasized the efforts of doctor and midwife training organizations to increase the accuracy and reliability of virginity testing, following several studies on virginity testing in forensic medical centers of Tehran and Shiraz (Tofighi Zavareh et al., 2002; Hedjazi, Zarenezhad, Shaykh Azadi, & Valie, 2012).

Virginity testing for cultural reasons has been declared as sexual violence against women by the World Health Organization (Zeyneloğlu, Kısa, & Yılmaz, 2013) and has been criticized as a patriarchal belief, gender inequality, and violent behavior against women (Christianson & Eriksson, 2011; Juth, & Lynöe, 2015; Awwad et al., 2013). Nevertheless, the use of virginity testing to assess a woman’s sexual activity is growing in the 21^st^ century (Wells, 2006); it is testing that, regardless of its result, causes harm to women (Hedjazi, Zarenezhad, Shaykh Azadi, & Valie, 2012).

In Iran, assessment illuminates the incompatibility of virginity testing with patient rights, particularly the necessity for respect of human dignity and privacy, the right to choose and decide freely, and the right to services based on respect for client privacy (Zahedi & Larijani, 2011).

According to declarations (Estafeta) obtained from the offices of Shiite authorities (Shi’a Mojtahids), Ayatollahs Husaini Sistani, Makarem Shirazi, Saafi Golpayegani, al-Fayyad, Alavi Gorgani, Mousavi Ardebili, Sobhani, Bayat Zanjani, Rouhani and Kabuli virginity testing is unlawful (haram), i.e. not permitted in the absence of a judicial and medical need (Husaini Sistani, 2014; Makarem Shirazi, 2014; Saafi Golpayegani, 2014; Al-Fayyad, 2014; Alavi Gorgani, 2014; Sobhani, 2014; Bayat-Zanjani, 2014; Mousavi Ardebili, 2014; Rohani, 2014; Kabuli, 2014).

Nevertheless, it is widely prevalent for cultural reasons in many Muslim countries, especially African and Asian countries (Khozan, 2013; Eşsizoğlu et al., 2011).

Tofighi-Zavareh and Hijazi reported that one of the most common causes of girls referring to forensic medical centers is virginity testing before marriage ((Tofighi Zavareh, Mosavipour, & Nematolahi, 2002; Hedjazi, Zarenezhad, Shaykh Azadi, & Valie, 2012). Despite inconsistency of virginity test and Iran patients’ right charter (Zahedi & Larijani, 2011) the test not only is performed by forensic medicine centers upon civil and criminal courts’ orders, but it also is performed by private clinics by physicians and midwives. According to Tofiqi Zavareh, 30.7% of virginity tests in forensic medicine centers were performed to check premarital relationship, 19.8% to check sexual violence, 18.8% to issue virginity certificate, 17.6% for divorce cases, 7.7% to issue recommendation to welfare centers; 1% to issue permission repair interventions; 2% to examine claimed events; and 2% to issue certificate of virginity for termination of marriage contract. (Tofiqi Zavareh et al., 2002)

The rate of premarital sex in Iran is increasing (Saliminia, Jazaieri, & Mohammadkhani, 2006), with prevalence rates reported as being 20% of sexual relations among students (Garmaroudi, Makarem, Alavi, & Abasi, 2010), 16.2% of premarital sex among male students of Qazvin (Simbar, Ramezani Tehrani, & Hashemi, 2003), 25% among female students in the big universities of Tehran (Khalajabadi Farahani, Cleland, & Mehyar, 2011), and 30% of sexual experience among Iranian boys aged 25 years and younger (Sharifi Saei, Isari, & Talebi, 2011). Based on these reports, virginity testing to assess the intactness of the hymen has caused an increase in unprotected oral and anal sex and subsequent increases in STDs and unplanned pregnancies (Simbar, Ramezani Tehrani, & Hashemi, 2003).

On the other hand the hymen is a thin mucous membrane; partially enclose the vaginal orifice which is not an suitable sign of virginity. It may be ruptured or not rupture by sexual intercourse (elastic hymen). Otherwise, it may be rupture accidentally by tampon use or through some exercise (Hegazi & Rukban, 2012) Acts of violence against women with damaged hymens prior to marriage is a significant matter that has been criticized by various international forums in many Mediterranean and Asian countries (Zeyneloğlu, Kısa, & Yılmaz, 2013; Cook & Dickens, 2009; Ahmadi, 2015), especially violence that leads to “honor killings.” These murders are not limited to Mediterranean countries, but are also reported from Bangladesh, Brazil, Egypt, India, Israel, Italy, Jordan, Pakistan, and Sweden (Zeyneloğlu, Kısa, & Yılmaz, 2013; Cook & Dickens, 2009). The increased demand for hymen repair surgery to prevent personal and social devastation as a direct result of loss of virginity has given the consequences of virginity testing a new dimension (Awwad et al., 2013).

The scarcity of available studies on this matter leaves health service delivery systems unaware of the complexity of the facts related to virginity testing and its overt and covert damages. Given that neglecting the negative consequences of virginity testing is considered a violation of the principle of respecting human rights (Gürsoy & Vural, 2003), this study explored the perception and experience of virginity testing examiners in forensic medicine centers and hospitals in Tehran.

## 2. Methods

### 2.1 Participants and Data Collection

This study, a qualitative research with interpretative phenomenological analysis, was conducted in 2014 in forensic medical centers and gynecology clinics of Tehran. To collect data, 16 in-depth semi-structured interviews were conducted with 11 physicians (5 Forensic medical specialists, 5 gynecologists, and 2 general physicians) and 4 midwives, all of whom were women with more than 10 years’ experience. A heterogeneous population of examiners from 6 forensic medical centers and several gynecology clinics of governmental hospitals in different areas of Tehran, which represented one-fifth of Iran’s population from different ethnic, race, economic, and social groups, participated in semi-structured in-depth interviews.

Participants were sampled purposively from hospitals and forensic medical centers, through face to face introduction following a snowball technique where one person would put the researcher in touch with her colleagues and other contacts who had virginity testing experience. Semi-structured interviews were conducted by the researcher (first author). As experienced in this study, some women who were introduced to the researcher refused to take part in the interview because of recording of interview. If they accepted to interview, they preferred to do it in a private space rather than in focus group discussion. Thus, only individual interviews were used to collect data in this study. The researcher explained the purposes of the research, that participation is voluntary and that they may stop the interview at any time and that confidentiality of records is ensured. Following their approval to participate in the interview, their consent, verbal or written, to record the interview was also obtained. Participants were asked to describe their experience of the virginity testing. A demographic questionnaire which was developed by the researchers was used in each interview. It included questions about age, experience, occupation, location and marital status. Interviews were based on topic guides, including a series of broad interview questions which the researcher considered to explore and probe with the interviewee. The questions that were used to guide the participants in the interviews include:

1) What does “virginity testing” feel like?
2) Base on your experience, what problems are about virginity testing?
3) What are the possible ways to improve the process, and situation regarding virginity testing?

It is ethically important to phrase questions in a manner that is not leading and that is open to participants’ ideas of what is most important (Callary, 2013).

Data collection was stopped when data saturation was reached, i.e. no new themes or ideas were being generated during the discussions. Interviews lasted 25 to 85 minutes, and second interviews were performed to confirm data and fill in possible gaps. All interviews were immediately transcribed and coded. Individual transcripts were sent to each participant to confirm they faithfully represented the conversation. No participants indicated problems with transcripts. Observational methods, recall, and records in the field were also used to gather, complete, and interpret the data.

### 2.2 Scientific Accuracy and Validity of Data

Evaluation criteria for Credibility, transferability, Conformability and Dependability of data was used to enhance the accuracy and consistency of the data (Lincoln, & Guba, 1985). To evaluate the credibility of the data, the member-checking method was used; analysis results were given to two members of the research team who added their complementary and critical comments. Handwritten interviews and extracted codes were given to the interviewees to verify that the text of the interview accurately reflected their experiences (Lincoln, & Guba, 1985). Participants and stages of the study were well-designated, and the opportunity to examine documentation was provided. Three professionals in the fields of qualitative research, reproduction health, and forensic medicine were asked to review the reports and transcripts and express their findings to define the similarity of the results. To assess the dependability of the data, the text of one interview was given to two researchers not involved in this study to determine if they achieved the same results.

### 2.3 Ethical Considerations

Before data collection, the necessary licenses and introductory letters to be presented to forensic medical centers and gynecology clinics were obtained from Shahid-Beheshti University of Medical Sciences. Written informed consent to interview and record them was obtained from all participants. The researcher explained the objectives of the study to participants before each interview and assured them that the study was voluntary and they could stop the interview and leave the research whenever they desired. All names were changed to maintain the anonymity and security of the interviewees. An Ethics Committee code was obtained from Shahid-Beheshti University of Medical Sciences for this trial.

### 2.4 Data Analysis

Smith’s method for interpretive phenomenology analysis was used to analyze data. Smith and Osborn noted that a meticulous case-by-case analysis of individual transcripts can be a lengthy process. So the analysis of qualitative data in this study followed a sequential manner, beginning with analysis at an individual-level (i.e., person-by-person) for each of the 16 individuals before proceeding to a group-level analysis that brought together data all 16 individuals.; at the end of each interview, it was important to put aside the coding from the previous transcripts in order to respect the convergences and divergences in the data (Smith, Flowers, & Larkin, 2013). As it is very difficult to “forget” what had coded in past transcripts. So the team analysis process was not without challenges.

The recorded data was typed in the shortest possible time, and the text was checked for semantics, language, and tone used by the participants. Then, each group of basic codes containing common sense and meaning was identified in one sub-category or indicated by one label or title. Although. The researcher searched appropriate themes and tried to map the internal relationships, connections, and patterns in the transcript to reduce detail. After analyzing each interview, the researcher began the analysis of the participant. It should be noted that during the study, the researcher tried to forget the ideas and themes of previous participants as much as possible.

In the final stage of analysis researcher listed all themes that were coded in each transcript, examined all themes’ operational definitions to find ones that were similar across all participants, and combined similar themes to provide a comprehensive and general model (Smith, 2011). Final coding included 5 themes, 13 main categories, and 31 sub-categories. Code management was performed by MAXQDA10 software.

## 3. Results

A total of 16 female examiners, all but one of whom had more than ten years’ experience in virginity testing and met the inclusion criteria, were interviewed. A second interview was needed with two of the participants, bringing the total number of interviews to 18. Demographic characteristics of the examiners are shown in Table 1, and themes extracted from the qualitative study are given in Table 2.

**Table 1:** Demographic characteristics of the examiners (n=16)

Marital Status	Years of experience	Age (Year)	Occupation	No
Married	12	38	Gynecologist	**1**
Married	20	48	Gynecologist	**2**
Married	8	38	Gynecologist	**3**
Married	27	60	Gynecologist	**4**
Married	12	45	Gynecologist	**5**
Married	15	47	Forensic medical specialist	**6**
Married	18	45	Forensic medical specialist	**7**
Married	15	44	Forensic medical specialist	**8**
Married	10	49	Forensic medical specialist	**9**
Married	13	38	Forensic medical specialist	**10**
Married	12	38	General Physicians	**11**
Married	12	38	General Physicians	**12**
Married	18	46	Midwife	**13**
Single	19	45	Midwife	**14**
Married	13	36	Midwife	**15**
Single	11	32	Midwife	**16**

**Table 2:** Themes extracted from the qualitative study (n=16)

Main-Categories	Sub-Theme	Theme
Individuals’ fear	Informal reasons	**Informal and formal reasons for virginity testing**
Social factors		
Medical necessity	Formal reasons
legal necessity	
Inadequate educating	Inadequate educating and poor performance	**Nature of virginity testing**
poor performance		
Test accuracy	Doubtful diagnosis	
Instructions		
Supporting examined cases	Supporting examined or honoring a professional pledge	
Honoring professional pledge		
The examiner’s assumption of the examinee’s point of view toward virginity testing	Views affecting the performance of virginity testing	
Attitude of examiners on performing virginity testing		
Beyond an examination		
Virginity	Beliefs	**Beliefs and cultural values underlying virginity testing**
Virginity testing and defloration		
Double standards	Cultural values	
Cultural changes		
Sexually transmitted diseases	Reproductive health threat of keeping hymen intact	**Overt and covert consequences of virginity testing**
Unwanted pregnancies		
Unusual sex		
Sexual dysfunction		
Individual consequences	Consequences of reporting damaged hymen	
Family		
The examiners		
Necessity of hymen repair	Ethical and legal implications of hymen repair	
Legal and ethical ambiguity of hymen repair		
Underlying cause of crime		
Maintaining patient rights	Promoting ethics and professional skills	**Optimizing service**
Improving education and research system		
Interaction with legislators	Interaction with legislators and people	
Interaction with people		

### 3.1 Informal and Formal Reasons for Virginity Testing

#### 3.1.1 Informal Reasons

***Individuals’ fear:*** Most participants pointed out that in Iran, virginity means having an intact hymen. They gave obtaining a virginity certificate before marriage and fear of hymen injury due to trauma, masturbation, or premarital sex as the main reasons for virginity testing among their clients. Obsessive virginity testing following actual or possible hymen injuries was mentioned by some participants as a reason for testing among Iranian women.

“I had a patient who referred to me once a week because of a midwife’s report that her hymen was not intact. She asked me to examine her again to see if her hymen was intact.” (Forensic medical specialist, 38 years old with 13 years’ experience)

Some participants reported that, because of the dominant belief that dilatable hymens will not be damaged; some clients visited them to check for this kind of hymen so they could have premarital sex without the fear of it tearing.

***Social factors:*** Although some participants indicated that traditional and religious people refer for virginity testing more than others, they agreed that virginity testing is conventionally requested because of cultural factors and social pressures by all people, even educated ones.

#### 3.1.2 Formal Causes

***Medical necessity:*** Some participants gave medical causes for virginity testing, namely the assessment of anatomical barriers of intercourse, hymen anomalies, especially imperforated hymens, and testing virginity before trans-vaginal or vestibular surgery.

“Single women undergoing surgery through the vagina come to get a virginity certificate.” (Forensic medical specialist, 49 years old with 10 years’ experience)

***Legal necessity:*** Assessing sexual assault and the accusation of not being a virgin, especially in the absence of wedding night bleeding, were given as legal reasons for virginity testing. According to some participants’ reports, virginity testing is necessary before girls can enter welfare centers for the prevention of sexual abuse.

“By law, if girls undergo virginity testing before entering welfare centers, they should be re-examined if they go out of the center.” (Forensic medical specialist, 49 years old with 10 years’ experience)

Given that a divorced Iranian woman can legally remove her ex-spouse’s name if her hymen is intact or can receive the full marriage portion if her hymen is damaged, these women comprise one group of applicants for virginity testing.

Because a virgin girl can be married only with the permission of her father or grandfather, girls who want to get married without permission also apply for virginity testing.

### 3.2 Nature of Virginity Testing

#### 3.2.1 Inadequate Educating and Poor Performance

***Inadequate educating:*** In this study, the unavailability of Persian and English resources, re-training courses, and appropriate educational practices, especially for midwives, were reported orally as evidence of the inappropriate quality of education.

“Not only are there no retraining courses, but the courses that are available are not suitable.” (Forensic medical specialist, 45 years old with 18 years’ experience).

***Poor performance:*** Some forensic specialists mentioned that the false reporting of damage to intact hymens by some midwives and gynecologists is inappropriate and expressed doubts about the technique and error of their own examination.

“I was in a city where gynecologists and midwives often reported intact hymens as damaged. Finally, I went before a judge and asked him to do something; they were ruining all the girls in the city.” (Forensic medical specialist, 38 years old with 13 years’ experience).

#### 3.2.2 Doubtful Diagnosis

***Test accuracy:*** Although most participants reported the results of examination doubtful because of the unreliability of virginity testing in diagnosing penetration and the reason for a damaged hymen, they were united in stating that an intact hymen does not indicate virginity, nor is a damaged hymen (in cases of trauma and abuse) a sign of lack of virginity. Only one participant insisted that the results of virginity testing of forensic medical centers were 100% accurate. “There are few cases that can be assuredly reported as intact.” (Gynecologist, 48 years old with 20 years’ experience).

***Instructions:*** Some participants pointed to the lack of guidelines related to qualifying individuals for examining, how to deal with offenders and virginity restoration applicants as committers of a crime, and the lack of legal validity of the certificates issued outside forensic medical centers as problems.

#### 3.2.3 Supporting Examined or Honoring A Professional Pledge

***Supporting examined cases:*** Some participants declared that for cases in which reporting a damaged hymen will threaten patients’ lives and ruin their futures, girls can be supported by providing them with a false certificate, not reporting the results of the examination, or writing ambiguous results of the examination.

“I examined a girl, and it was obvious that her hymen was not intact. If I were to state this, they would definitely kill me; so, I gave her a certificate stating that she had had no sexual relations.” (Gynecologist, 45 years old with 12 years’ experience).

***Honoring professional pledge:*** Some participants stated that making any false reports, even in the face of serious threats to the examined patient, is a crime and not acceptable, while emphasizing the importance of honoring one’s professional pledge and faithfulness to medical ethics.

Contrary to the belief of gynecologists, midwives, and general physicians that forensic medical specialists are lenient in reporting cases of a damaged hymen, most forensic medical specialists participating in the current study emphasized the professional pledge and stated dishonest reports are contradictory to medical ethics and a crime, even when serious threats exist for the examined patient.

“We cannot help even if the woman’s life is under threat and cannot give a false certificate, as it is a crime.” (Forensic medical specialist, 38 years old with 12 years’ experience).

#### 3.2.4 Views Affecting the Performance of Virginity Testing

***The examiner’s assumption of the examinee’s point of view toward virginity testing***: Except for one participant who stated that virginity testing is as easy as examining a patient’s throat, others reported virginity testing was not only an invasion of privacy, but also spiritually and physically difficult. Such feelings cause a deterioration of the patients’ psychological distress, especially in cases of a medical commission.

***Attitude of examiners on performing virginity testing:*** Except for one participant who preferred to do the virginity testing to increase talent and awareness, all others reported different stages of unwillingness to perform virginity testing, because of its negative consequences and its cause a psychological distress.

“I do not like anyone to come to my clinic for virginity testing.” (Gynecologist, 48 years old with 20 years’ experience).

***Beyond an examination:*** Especially for child victims of sexual assault, mentally disabled persons, cases of incest, and cases in which reporting the results is life-threatening for the patient, virginity testing is more than just a medical examination.

“An eight-year-old girl was suspected of being sexually abused. Her grandmother and father tried to hold her hands still, but they couldn’t. The kid was shouting and crying, and I was going to burst into tears.” (General physician, 38 years old with 12 years’ experience).

### 3.3 Beliefs and Cultural Values Underlying Virginity Examination

#### 3.3.1 Beliefs

***Virginity and Defloration:*** Deep-rooted false beliefs, such as wedding night bleeding is a symbol of virginity, even in the educated class, were the focus of the current study. In this context, following the belief of losing life in the case of a damaged hymen, defloration due to sexual assault was reported as more disturbing than the physical and psychological damage caused victims of rape.

“If the hymen is damaged, the girl thinks she has lost everything.” (Gynecologist, 48 years old with 20 years’ experience).

***Virginity Testing:*** Some participants stated the view that reports of intact hymens show loyalty to ethical and religious principles. Moreover, because of the belief that dilatable hymens do not hurt during intercourse, virginity testing was defined as a cause to take hymen-associated stress away.

#### 3.3.2 Cultural Values

***Double standards:*** Although most examiners considered premarital virginity testing an insult to the girl’s intelligence, her personality, and as a cause of gender inequality, they also expressed the belief that virginity testing would never be performed if the virginity testing of men was practical. Most participants advised premarital virginity testing for their female relatives because of the social vulnerability of Iranian girls.

“For my daughter, I will take the certificate.” Gynecologist, 60 years old with 27 years’ experience).

***Cultural changes:*** An increase in the rate of premarital sex was mentioned as a reason for covert premarital virginity testing, increases in the number of clients requesting virginity testing (particularly single clients), the reduced age of clients referring for virginity testing, and the reduction in the severity of violence towards women in the event of hymen injury.

### 3.4 Overt and Covert Consequences of Virginity Testing

#### 3.4.1 Reproductive Health Threat of Keeping Hymen Intact

***STDs:*** Most participants reported that an increase in unprotected genital contact caused the increased rates of genital warts, herpes, AIDS, and unwanted pregnancy in patients.

***Unwanted Pregnancies:*** Unwanted pregnancy occurring after unprotected genitalia contact and anal sex was also introduced by some participants as a result of virginity testing.

***Sexual dysfunction:*** Only two participants in the current study expressed that a fear of damaging the hymen is a cause of sexual dysfunction, especially vaginismus.

***Unusual Sex:*** Some examiners mentioned hymen damage caused by unusual sex as a negative consequence of keeping the hymen intact.

“A girl has oral and anal relationship so as to keep her hymen intact. She feels proud that she has maintained her virginity; meanwhile, she has no idea that she will get fecal incontinence in the future.” (Forensic medical specialist, 49 years old with 10 years’ experience).

#### 3.4.2 Consequences of Reporting Damaged Hymen

***Individual consequences:*** Most participants defined rejection, suicide, depression, weakened self-confidence, run-outs, and divorce, as well as an increased risk of the diversion and abuse of girls as individual consequences of virginity testing in their clients.

“I told her that her hymen was not intact, and she said that she had done nothing. Then I heard she had committed suicide.” (Gynecologist, 38 years old with 12 years’ experience).

***Family:*** Ethnic conflicts, putting the family prestige in danger, and even murder were mentioned as consequences of virginity testing.

***The examiners:*** Two participants reported that single girls insist on vaginal examinations by a doctor to hide that their hymen has been damaged by intercourse; the examiner is bothered by the negative consequences of reporting a damaged hymen.

#### 3.4.3 Ethical and Legal Implications of Hymen Repair

***Necessity of hymen repair:*** While emphasizing the necessity of hymen repair in cases of trauma, rape, or vaginal surgery, some participants referred to hymen repair as a way to prevent the diversion of girls and divorce in cases of hymen injury.

Legal and ethical ambiguity of hymen repair: The lack of instructions regarding hymen repair and the uncertainty of its legal and ethical status were also mentioned in this study.

“Is the one who repairs a damaged hymen committing a criminal or solving family problems? This needs to be clarified.” (Forensic medical specialist, 44 years old with 15 years’ experience).

***Underlying cause of crime:*** Some participants declared that hymen repair is illegal and a crime, like illegal abortion, for reasons such as the possibility of abuse from the examined patient, the receipt of exorbitant fees, damage to the genital system caused by non-experts, and trick in marriage.

### 3.5 Optimizing Service

#### 3.5.1 Promoting Ethics and Professional Skills

***Maintaining Patient Rights:*** The necessity of permission for testing and reporting the results to others, respecting the examined cases regardless of the reason of testing and its results, and training the examined cases were unitedly reported in the present study. One participant pointed out the necessity of determining an age range for virginity testing, considering testing children staying at welfare institutes.

“If parents want to know the results of virginity testing, I’ll tell them if the girl permits me; otherwise, I will not tell them anything.” (Forensic medical specialist, 38years old with 13 years’ experience).

***Improving Education and Research System:*** Some participants emphasized the need to learn how to deal with cases of damaged hymen, especially those damaged by sexual abuse, and the need for continuous training courses. Only one of the forensic medical specialists suggested research to update examination techniques, draft guidelines, and determine the use of modern technology and paraclinic methods for testing. However, all participating forensic medical specialists agreed that training courses and certification are needed for qualifying individuals for virginity testing.

#### 3.5.2 Interaction With Legislators and People

***Interaction with legislators:*** Examiners emphasized the need for hymen repair in cases of rape, genital trauma, and surgical procedures. Although most participating forensic medical specialists reported that virginity testing in clinics should be illegal, participating gynecologists and midwives emphasized the legal validity of certificates affirmed in clinics.

One participant stated that virginity testing should be declared illegal, while another declared that outlawing virginity testing would lead to a violation of women’s rights.

Some participants in this study stated that virginity testing should not be allowed in forensic centers, because of its being scary, the risk of being labeled, the lack of reporting of results to patients, the risk of tissue damage, and the court permission that is required. Conversely, some participants felt that forensic medical centers are ideal places for virginity testing, because of fewer testing errors, the legal validity of certificates issued, and lower testing costs.

***Interaction With People:*** All participants agreed that educating people that obtaining proof of virginity on the wedding night or obtaining a virginity certificate is unnecessary and strengthening an appropriate parent-child relationship testing is more effective in preventing virginity testing and its consequences than legal approaches. Moreover, there was a consensus on the necessity of strengthening religious and cultural beliefs regarding virginity pledges.

“The cultural and social values must change so that a girl’s virginity is not judged by an intact hymen.” (Gynecologist, 48 years old with 20 years’ experience).

## 4. Discussion

### 4.1 Discussion of Results

Qualitative studies are particularly valuable for investigating complex and sensitive issues such as virginity (Zeyneloğlu, Kısa, & Yılmaz, 2013). The current qualitative study is the first one conducted in Iran that investigates what Iranian examiners have perceived and experienced through virginity testing. The importance of qualitative studies lies not in the generalizability of the results, but in the correct description of an experience which leads to clarification and deeper thought about the phenomenon (Green & Holloway, 1997). The current study explained the perception and experience of virginity testing in five themes.

Results of this study indicated that individual and social factors are informal causes of the virginity testing of Iranian women. Gürsoy claimed the illegality of virginity testing because of individual and social reasons such as proof of premarital virginity, lack of wedding night bleeding, remarriage, trauma, or assessment of premarital sexual activity and named virginity testing as a social problem in Turkey (Gürsoy & Vural, 2003). Requests for virginity certificates and hymen repair among Sweden immigrants (Juth, Tännsjö, Hansson, & Lynöe, 2013) and routine virginity testing in South Africa for cultural reasons (Khozan, 2013; Taylor et al., 2007) also confirm the findings of this study. African girls are reported to be willing to undergo virginity testing to maintain cultural values, but this and other studies have shown testing to be obligatory because of social pressures, which seem to be caused by differences in beliefs and cultural values among these societies.

The current study found the formal reasons of virginity testing being an obligatory examination in forms of medical and judiciary necessity. Shalhoub claims cases of consensual sexual relations with girls under the legal age, divorce, hymen injury reports, lack of bleeding during the first intercourse, and obtaining premarital virginity certificate, which are covered by medical and judiciary necessity (Shalhoub Kevorkian, 2005; Shalhoub Kevorkian, 2000; Cindoglu, 1997).

Turkey, however, has declared virginity testing for the above-mentioned reasons illegal (Zeyneloğlu, Kısa, & Yılmaz, 2013; Gürsoy & Vural, 2003). It seems that the approaches to virginity testing of service provider systems in various countries are the main causes of these differences

Insufficient education and performance and unreliability of virginity testing in diagnosing virginity were found in this study as well as in other studies (Gürsoy, & Vural, 2003). In this regard, Cook (2009) and Amy (2008) stated that there is no valid medical testing to prove that a woman is virgin, despite the fact that some doctors, particularly in Asia and South Africa, support virginity testing in diagnosing virginity (Juth, Tännsjö, Hansson, & Lynöe, 2013). It seems that false beliefs held even by the educated class have caused inconsistent reports in this area.

The results of the current study regarding the lack of instructions related to virginity testing as a challenge correlate with the need for guidelines of virginity and hymen repair certification in Sweden (Juth & Lynöe, 2015). Some studies, however, have emphasized the inadequacy of the guidelines while mentioning the inefficiency of the regulations, such as outlawing virginity testing in Turkey (Gürsoy & Vural, 2003) and banning testing girls under 16 years in Africa (Khozan, 2013; Taylor et al., 2007).

The findings of this study support the examined patients while maintaining a professional pledge, which can also be seen in other studies. Palestinian doctors agree on the inscription of false certification to support examined patients (Shalhoub Kevorkian, 2005). Reports claim the unnecessariness of implementing medical ethics in cases where life is threatened, despite disapproving of virginity certification (Juth, Tännsjö, Hansson, & Lynöe, 2013).

Another finding of the present study is the difficulty of virginity testing. Other studies have reported this problem as being displayed in a variety of forms, such as screaming, crying, being uncooperative, pushing, and freezing (Shalhoub Kevorkian, 2005). Virginity testing is described as a dishonorable experience for women (Gürsoy & Vural, 2003). Conversely, the results of some studies from Africa report that families celebrate the occasion of receiving a virginity certificate, which represents the willingness of African girls to view virginity testing as a pleasant and proud experience (Khozan, 2013; Taylor et al., 2007). It seems that cultural factors and undeniable differences in cultural values in different societies cause different reports regarding how virginity testing is viewed.

Cultural values and beliefs underlying virginity testing, even among the educated class, were part of the findings of this study and were described in other studies as influencing the importance of an intact hymen in diagnosing virginity, even among health professionals (Juth, Tännsjö, Hansson, & Lynöe, 2013). A study in Turkey mentioned that some midwives and nurses advise grooms to require virginity testing from their brides before marriage; a university education does not make a difference in these beliefs (Gürsoy & Vural, 2003).

The erroneous belief of wedding night bleeding as a symbol of virginity was one of the findings of the present study that was referred to in other studies as delivering the bloody stain sheet to prove virginity. Since Asian doctors support the belief of wedding night bleeding, some Asian migrants to European countries who wished to return to their homeland applied for a virginity certificate or hymen repair (Juth, Tännsjö, Hansson, & Lynöe, 2013), despite the fact that, according to available documents, only some girls bleed in their first intercourse (Zeyneloğlu, Kısa, & Yılmaz, 2013; Juth, Tännsjö, Hansson, & Lynöe, 2013).

Having unprotected oral or anal relations to prevent vaginal intercourse as a consequence of virginity testing, mentioned as a cause of defeat of the virginity pledge in the United States, is also consistent with our results that showed such relations cause an increase in STDs (Antoinette, Landor, & Gordon, 2013; Melina et al., 2007). Meanwhile, virginity testing is largely performed as an approach to preventing STDs and teen pregnancies in African countries (Khozan, 2013; Taylor et al., 2007).

Suicide, murders, weakening self-confidence, and psychological problems were introduced in the present study and many others as consequences of virginity testing, especially in Turkey (Gürsoy & Vural, 2003; Juth, Tännsjö, Hansson, & Lynöe, 2013; Shalhoub Kevorkian, 2005). In spite of the consensus of most studies that suicide is a consequence of virginity testing in cases of damaged hymens, reports of suicide because of obligatory virginity testing (regardless of its result) were seen only in Turkey. Except cultural issues, no other justification seems to explain this exception

The findings of this study regarding sexual dysfunction as a consequence of virginity testing are also mentioned in other studies (Gürsoy & Vural, 2003).

The consequence of sexual abuse by male doctors was not reported in this study, but Shalhoub (2005) pointed out the consequences of sexual abuse and rape of examined women by male doctors and declared that emotional, physical, and social trauma is not a negligible consequence of virginity testing.

The consequence of forced marriage in victims of sexual assault with abusers following the report of a damaged hymen was also reported in some studies, but not in our study (Shalhoub Kevorkian, 2005).

Reports of damage to the genital system following hymen repair by unprofessional individuals was also pointed out in this and other studies (Ahmadi, 2015) in the form of infection and other complications of hidden hymenoplasty by non-doctor professionals (Juth, Tännsjö, Hansson, & Lynöe, 2013).

Our finding of making trouble for examiners by deceiving the doctor and covering the reason for a damaged hymen was not reported in other studies. Some studies did mention the preference of doctors to refer damaged hymens to other centers to protect themselves, their children, and their clinic as a consequence of disturbance for the examiner (Shalhoub Kevorkian, 2005).

Obtaining consent from the examined patient to perform virginity testing was a finding of this study that was mentioned in most studies as necessary (Gürsoy & Vural, 2003). Educating the patient and preserving their personal privacy were also found in this study and were mentioned in other studies with an emphasis on patient education for testing and covering her during the testing (Gürsoy & Vural, 2003).

The results of Kandiyoti’s study regarding training examiners for confronting special cases, such as rape victims, children, and alcoholic patients (Kandiyoti, 1987), are similar to the results of this study; however, contrary to the expectations of the researcher, the necessity of referring sexual assault victims to specialized centers for help with social, psychological, physical, and legal problems mentioned in Martin’s study was not mentioned in the current study (Martin, Di Nitto, Maxwell, & Norton, 1985). Examiners in the current study agreed on the need to repair the hymen in cases of trauma and sexual assault which was highlighted in several studies as a means to providing conditions for a good marriage (Gürsoy & Vural, 2003).

The findings of this study regarding the importance of improving cultural and religious beliefs in order to avoid the consequences of virginity testing and unnecessary examination was proposed in other studies as a necessary education for social change and a lack of efficiency in legal regulations for solving virginity problems (Gürsoy & Vural, 2003). Meanwhile, results of Antoinette’s study regarding the positive role of religion in delaying the start of sexual relations in individuals that have kept their virginity based on a virginity pledge (Antoinette, Landor, & Gordon, 2013) were also consistent with the results of the current study regarding improving religious beliefs to maintain the virginity pledge.

### 4.2 Limitations

The unwillingness of some examiners to participate in the study was a limitation of this study, as they might have had different experiences with virginity testing.

### 4.3 Strengths and Weaknesses of the Study

One strength of this study is the contribution of forensic medical specialists’ completely unique experiences as the most professional group involved in virginity testing. One limitation of this study is the lack of participating by male doctors. Since virginity testing is not routinely performed by male doctors in Iran, male doctors with virginity testing experience were not included, even though they were assured that their identify would be kept confidential. Nothing exists to indicate whether or not male doctors are authorized to carry out such examinations. Perhaps if there were clear instructions in this regard, they would accept a researcher’s invitation to participate in a study.

### 4.4 Suggestions

Considering the effective role of social and cultural factors on virginity testing, more studies are needed to compare virginity testing phenomena with greater focus on its causes and consequences in patients and examiners of various ethnic and racial groups. Moreover, for a more complete review of all aspects of the virginity phenomena, qualitative research including examined patients, examiners, patients’ families, and judicial authorities as the key participants is recommended.

## 5. Conclusion

The current study found that, considering cultural factors that affect the performance and overt and covert consequences of virginity testing, it is performed in Iran for formal and informal reasons beyond a medical examination as a social product. Furthermore, it is perceived with ambiguity regarding its accuracy and certainty of examination and uncertainty about the ethics and reproductive rights regarding virginity testing by the examiner. Examiners are affected by the nature of the examination, overt and covert harms of virginity testing, beliefs and values underlying virginity testing, and formal and informal reasons for the examination
